# Commentary: Cognitive behavioural therapy for the treatment of chronic fatigue syndrome in adults: a short analysis of the meta-analysis

**DOI:** 10.3389/fpsyt.2025.1746712

**Published:** 2026-03-27

**Authors:** Mark Vink, Friso Vink-Niese

**Affiliations:** 1 Independent Researcher, Amsterdam, Netherlands; 2 Independent Researcher, Osnabrück, Germany

**Keywords:** CBT, chronic fatigue syndrome, cognitive behavioural therapy, fatigue, ME/CFS, meta-analysis, myalgic encephalomyelitis, physical functioning

## 1 Introduction

The meta-analysis by Kolala et al. (1) selected 12 studies and examined whether non-protocol-based cognitive behavioural therapy (CBT) is effective in myalgic encephalomyelitis/chronic fatigue syndrome (ME/CFS) patients who have not been selected in accordance with the Oxford criteria. They chose this approach because experts have recommended that the Oxford criteria should not be used anymore because post-exertional malaise (PEM), the main characteristic of the disease, is not required for diagnosis according to these criteria. The primary outcome of the meta-analysis was fatigue, and the authors concluded that CBT did not lead to statistically significant improvement. Nevertheless, at the same time, they concluded that individual face-to-face CBT had a large effect on reducing fatigue and that self-directed CBT had a large effect on increasing physical functioning. None of their other analyses yielded statistically significant results. They also concluded that self-directed CBT should be used for patients with milder symptoms and that booster sessions may be required because of the lack of long-term efficacy of CBT. However, in a poster presentation for the Royal Australian and New Zealand College of Psychiatrists, they stated that CBT should be offered to all patients with ME/CFS (2). The British National Institute for Health and Care Excellence (NICE) reviewed the literature as part of the process to update its ME/CFS guideline and concluded that CBT studies were all of low or very low quality. NICE (3) also concluded that CBT does not lead to improvement or recovery. In this article, we analysed the evidence that was used by Kolala et al. (1) to come to their conclusions. Our analysis, however, shows that the data support the conclusions by NICE and that it does not support the conclusions of the meta-analysis. Moreover, Kolala et al. ignored the problems of the studies in their analysis.

## 2 Discussion

### 2.1 Registration

According to the meta-analysis’s registration with PROSPERO, the international prospective register of systematic reviews, the objectives of the review were to find out what the efficacy of CBT is on the quality of life and on fatigue in patients with ME/CFS at the end of treatment, as well as at long-term follow-up. According to the registration, articles that used the Oxford criteria will be excluded (4). However, there is nothing in the registration about determining the efficacy of individual face-to-face or self-directed CBT separately. Consequently, these are *post-hoc* analyses and deviations from their registration with PROSPERO. Why the meta-analysis did not mention that or why these *post-hoc* analyses were carried out is unclear.

### 2.2 The two objectives according to the registration

#### 2.2.1 Fatigue

Five studies using protocol-based CBT with a time-contingent increase in physical activity were used to claim that individual face-to-face CBT is effective for fatigue. However, these five studies, just like all the other studies in the meta-analysis, should have been excluded because they did not examine *non-protocol-based CBT*, which the meta-analysis said it would examine.

As noted by the meta-analysis, Prins et al. (5) used the Fukuda criteria, apart from requiring the presence of four of the eight additional symptoms. Consequently, they used the Oxford criteria and not the Fukuda criteria (6, 7). According to Deale’s long-term follow-up article (8), that study also used the Oxford criteria. Both studies should therefore have been excluded.

Jason et al. (9) concluded that there were few differential results among the four non-pharmacologic interventions (CBT, cognitive therapy with pacing, aerobic treatment, and relaxation). They also noted that their sample was not representative of ME/CFS because many patients were working, and their study was small.

Of the patients who were selected by Gotaas et al. (10), 25% (82/328) refused to take part in that study. This affects the generalisability of results of a study because people who think they will not benefit or are made worse by a treatment are usually the ones who refuse to take part in a study (11). In the 16-week CBT group, 24.4% (19/78) were not adequately treated (10), and in the 8-week interpersonal-oriented CBT group, this was 20.5% (16/78). In Prins et al. (5), 40.9% dropped out of the CBT group. According to Lilienfeld et al. (11), people who do not improve and/or are negatively affected by treatment are the ones who are most likely to drop out. Consequently, the remaining patients in such a study are not representative of a patient group as a whole. According to the 5 and 20 rule, studies are highly biased if 20% or more drop out or are inadequately treated (12). Consequently, both Gotaas et al. (10) and Prins et al. (5) were highly biased because of that.

The bias in both studies increased further because Gotaas et al. (10) did not publish their objective outcome measure (VO2max), and Prins et al. (5) did not publish two of them (objective cognitive function and the actometer). These two objective outcomes were published 6 and 9 years later, respectively, and their results showed that CBT does not lead to objective improvement (13, 14). Prins et al. (5) claimed that the absence of effect on their third objective outcome, the number of hours worked, was because patients did not have time to find work, even though they had 6 months or more to obtain employment. Additionally, the study of Van der Schaaf et al. (15) included female patients only, so it does not provide any evidence for the treatment of male patients. The Checklist Individual Strength (CIS)-fatigue score after CBT was 33.6, which is only slightly better than the score for severe fatigue (≥35). Moreover, a score of 30.5 is high according to a study by Korenromp et al. (16), which also found that patients with sarcoidosis in clinical remission had a score of 17.2 (16). This is similar to the score of the healthy controls in Van der Schaaf et al. (15), who had a score of 16.9. The scores after CBT in Prins et al. (5) were 40.5 at the end of treatment (8 months from baseline) and 39.5 at 6 months of follow-up (14 months from baseline). The other three studies did not use this scale. Consequently, patients are still very or severely fatigued after treatment with individual face-to-face CBT.

The Sickness Impact Profile-8 (SIP8) score after CBT in Van der Schaaf et al. (15) was 943.8, whereas with a score of ≥700, one was severely disabled according to the study itself, and the score of healthy women was 65.5 (17). After CBT in Prins et al. (5), the SIP8 score was 1,220 (end of treatment) and 1,170 (6 months of follow-up). These SIP8 scores show that patients were still severely disabled after treatment with individual face-to-face CBT. The other three studies did not use the SIP8 scale.

#### 2.2.2 Quality of life

Quality of life was one of the two objectives of the study to determine the efficacy of CBT (4). Kolala et al. (1) concluded that CBT has no statistically significant effects on the quality of life.

### 2.3 Physical functioning

Three studies were used to claim that self-directed CBT is effective for physical functioning. Janse et al. (18) noted that it is difficult to find an effect of internet-based CBT (iCBT) on physical functioning because their study did not have an entry requirement for physical functioning. Knoop et al. (19) did not use a physical functioning entry requirement either, and in Tummers et al. (20), one needed to have a physical and/or social functioning score of 70 or less to be included in the study. Consequently, none of the three studies had a physical function entry requirement, and therefore, none of the three studies should have been used to determine the efficacy of self-directed CBT for physical functioning.

According to Tummers et al. (20), a physical functioning score of 70 or less was a possible requirement to be eligible for the study. It also represents severe disability according to that study, and the score after treatment with self-directed CBT was only 65.4; consequently, patients were still ill enough to re-enter the same study and be treated with the same treatment again. Moreover, it also means that they were still severely disabled after treatment with self-directed CBT.

The SIP8 score after treatment in Knoop et al. (19) was 1,079; in Janse et al. (18), these scores were 867.8 (protocol-driven feedback iCBT) and 885 (feedback on demand iCBT); and Tummers et al. (20) did not use that scale. These SIP8 scores also show that patients were still severely disabled after treatment with self-directed CBT.

A basic requirement of systematic reviews and meta-analyses is that they should include all eligible evidence, but they should also encompass studies from different centres to establish robust evidence (21). Nevertheless, the three studies that were used to claim that self-directed CBT is effective for physical functioning were all carried out by the same institute, i.e. The Dutch Knowledge Centre of Chronic Fatigue [Nederlands Kenniscentrum Chronische Vermoeidheid or (NKCV)], whose director (Knoop) was involved in all three studies (22). Consequently, a conclusion about the efficacy of a treatment should not be based on studies from one institute only, even more so because those three studies may have been hampered by a financial conflict of interest because of the involvement of the director of the NKCV in all three of them. Why Kolala et al. (1) ignored all of that is unclear.

Finally, according to the NKCV, there is a bad correlation between subjective and objective measures of physical functioning. Most CBT studies do not use objective outcomes, but three studies by the NKCV that did use them showed that CBT does not lead to the objective improvement of physical functioning according to the actometer (14).

### 2.4 Criteria used

Kolala et al. (1) excluded studies that used the Oxford criteria because PEM, the main characteristic of ME/CFS, is not compulsory for diagnosis. Why they did not exclude the aforementioned two studies that used the Oxford criteria is unclear. Kolala et al. did not exclude studies that used the Fukuda criteria, even though PEM is only an optional criterion according to those criteria (7). The problem with the Fukuda criteria is that they lead to the inclusion of patients who do not have ME/CFS. Why the meta-analysis did not exclude these studies, or highlight this problem, is unclear.

### 2.5 Control group

A basic principle of a properly conducted study is that patients in the control group receive the same number of sessions, attention, and expectation raised about the efficacy of the control treatment as patients in the therapy group, so that the only difference between those groups is the therapy (23). Only then does one know that an improvement is due to the treatment and that it is not caused by all sorts of undefined differences between the groups. However, this was ignored by all three studies that were used to claim efficacy on physical functioning, as they used a waiting list control group. Two of the five studies that were used to claim efficacy on fatigue (10, 15) used a waiting list control group, and Prins et al. (5) used a natural course control group. Only Deale et al. (24) and Jason et al. (9) used an active control group (relaxation).

According to a study by psychology professor Cuijpers (23) entitled “How to prove that your therapy is effective, even when it is not: a guideline”, one can obtain false-positive results for an intervention using subjective primary outcomes in non-blinded studies. According to the same article, one can increase the chances of this happening by also using an inactive control group. Moreover, a systematic review by Fordham et al. (25), which concluded that CBT is effective for a range of diseases, including ME/CFS, also noted that if studies use an active instead of a passive control group, then the effect size is trivial (0.09).

### 2.6 Adverse effects

The meta-analysis concluded that many studies did not report adverse effects as an outcome, but serious adverse effects were not reported (1). The latter means that no one had to be admitted to hospital or died as a consequence of the therapy, which is not surprising because patients do not complain about this. Núñez et al. (26), one of the 12 included studies, did report about adverse effects and concluded that treatment had a negative effect on physical function and pain. They also concluded that treatment was ineffective and that it may be harmful.

### 2.7 Risk of bias

The meta-analysis used the Cochrane Risk of Bias Tool (RoB; 27). Of the eight studies that were used to conclude that CBT is effective for fatigue and physical functioning, only the study by Tummers et al. (20) was deemed to be at high risk of bias. There was some concern about the risk of bias in the selection of reported outcome in the study by Prins et al. (5). Nevertheless, as we have seen, this study did not publish two of its objective outcome measures, which showed that CBT did not lead to objective improvement, and they used a fallacy to ignore the null effect on the third objective outcome (number of hours worked). Moreover, 40.9% dropped out of the treatment group.

As we have seen in Gotaas et al. (10), 25% of participants who were selected refused to take part. In Janse et al. (18), the supplement shows that 81% (65/80, feedback on demand) and 84% (67/80, protocol-driven feedback) did not adhere to treatment in the two treatment groups. Consequently, these three studies should have been labelled as having a high risk of bias, too. Additionally, all studies were non-blinded by definition, which relied on subjective outcomes in studies where patients were instructed to interpret their symptoms differently, i.e. as symptoms of normal life instead of a disease. Consequently, one does not know if a change in scores in subjective outcomes is due to the treatment or if patients were simply interpreting questions at the end of treatment differently than at the beginning of the study. This is known as response shift bias (27), and the only way to correct for it in non-blinded studies is by using objective outcome measures (11, 28).

According to the Cochrane Risk of Bias Tool (29), it is important for review authors to judge whether the knowledge of the intervention received may have influenced participants’ reporting of an outcome. If that is deemed to be the case, then the risk of bias is high. Cochrane also gives an example of patients who had to report about pain in a study that compared acupuncture with no treatment. They concluded that it is likely that the knowledge of the intervention received influenced the reporting of the outcomes (29). Consequently, the studies in the meta-analysis had a high risk of bias.

## 3 Conclusion

This short analysis shows that there are many issues with the meta-analysis by Kolala et al. (1) and the studies in it. The meta-analysis was set up to examine whether non-protocol-based CBT was effective for ME/CFS in studies that did not use the Oxford criteria. Nevertheless, two of the included studies used these criteria, and none of the included studies examined non-protocol-based CBT. Moreover, PEM, the main characteristic of the disease, was not required for diagnosis in any of the included studies.

Kolala et al. concluded that CBT was not effective, but then used two *post-hoc* analyses to state that individual face-to-face CBT was effective for fatigue and that self-directed CBT was effective for physical functioning. However, even after those treatments, patients remained severely ill and severely disabled. Also, quality of life, which was one of the two objectives according to the registration of the meta-analysis, did not improve. Different forms of CBT did not lead to objective improvement, and an extensive review of the literature found that CBT has a negative instead of a positive effect on work and illness benefit status (30).

Only one study was deemed to be high risk of bias by Kolala et al. (1). Nevertheless, all studies were non-blinded studies that relied on subjective primary outcomes. Ten of the 12 studies used passive control groups, and a systematic review by Fordham et al. (25) found that the effect size of CBT is trivial if studies use an active control group instead of a passive control group. Adherence to treatment was bad, dropout was high, and studies used selective reporting of objective outcomes. Consequently, the risk of bias in the included studies was high.

In conclusion, our analysis confirms the conclusion by NICE (3) that the quality of CBT studies is (very) low and that CBT, irrespective of the form that is used, is not an effective treatment for ME/CFS.
